# A library of Neo Open Reading Frame peptides (NOPs) as a sustainable resource of common neoantigens in up to 50% of cancer patients

**DOI:** 10.1038/s41598-019-42729-2

**Published:** 2019-04-29

**Authors:** Jan Koster, Ronald H. A. Plasterk

**Affiliations:** 1 Amsterdam UMC, University of Amsterdam, Department of Oncogenomics, Meibergdreef 9, Amsterdam, The Netherlands; 2 myTomorrows, Antoni Fokkerweg 61, Amsterdam, The Netherlands; 3 Founder/CEO, Frame Cancer Therapeutics, Science Park 106, Amsterdam, 1098 XG The Netherlands; 4 Amsterdam UMC, Department of Clinical Epidemiology, Biostatistics and Bioinformatics, Amsterdam, The Netherlands

**Keywords:** Tumour immunology, Data mining, Peptide vaccines

## Abstract

Somatic mutations in cancer can result in neoantigens against which patients can be vaccinated. The quest for tumor specific neoantigens has yielded no targets that are common to all tumors, yet foreign to healthy cells. Single base pair substitutions (SNVs) at best can alter 1 amino acid which can result in a neoantigen; with the exception of rare site-specific oncogenic driver mutations (such as RAS) such mutations are private. Here, we describe a source of common neoantigens induced by frame shift mutations, based on analysis of 10,186 TCGA tumor samples. We find that these frame shift mutations can produce long neoantigens. These are completely new to the body, and indeed recent evidence suggests that frame shifts can be highly immunogenic. We report that many different frame shift mutations converge to the same small set of 3′ neo open reading frame peptides (NOPs), all encoded by the Neo-ORFeome. We find that a fixed set of only 1,244 neo-peptides in as much as 30% of all TCGA cancer patients. For some tumor classes this is higher; e.g. for colon and cervical cancer, peptides derived from only ten genes (saturated at 90 peptides) can be applied to 39% of all patients. 50% of all TCGA patients can be achieved at saturation (using all those peptides in the library found more than once). A pre-fabricated library of vaccines (peptide, RNA or DNA) based on this set can provide off the shelf, quality certified, ‘personalized’ vaccines within hours, saving months of vaccine preparation. This is crucial for critically ill cancer patients with short average survival expectancy after diagnosis.

## Introduction

The concept of utilizing the immune system to battle cancer is very attractive and studied extensively. Indeed, neoantigens can result from somatic mutations, against which patients can be vaccinated^1–11^. Recent evidence suggests that frame shift mutations, that result in peptides which are completely new to the body, can be highly immunogenic^12–15^. The immune response to neoantigen vaccination, including the possible predictive value of epitope selection has been studied in great detail^8,13,16–21^, and there is no doubt about the promise of neoantigen-directed immunotherapy. The quest for common antigens, however, has been disappointing, since virtually all mutations are private. One can derive algorithms that predict likely good epitopes, but still every case is different. Here we report that frame shift mutations, which are also mostly unique among patients and tumors, nevertheless converge to neo open reading frame peptides (NOPs) from their translation products, that result in common neoantigens in large groups of cancer patients.

**Figure 1:**
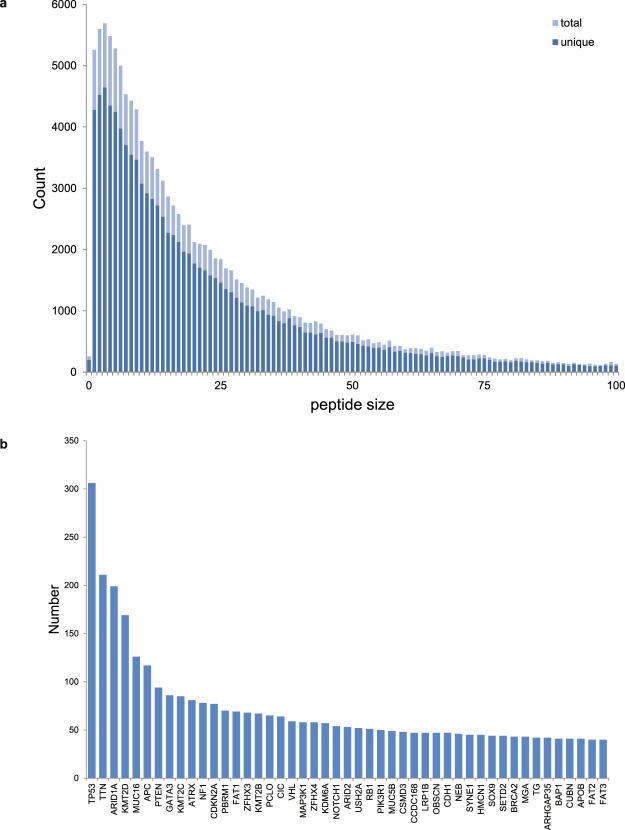
Frame shift initiated translation in the TCGA (n = 10,186) cohort is of sufficient size for immune presentation. (**a**) Peptide length distribution of frame shift mutation initiated translation up to the first encountered stop codon. Dark shades are unique FS mutations, light shade indicates the total sum (unique FS multiplied by number of patients containing that FS). (**b**) Gene distribution of peptides with length 10 or longer and encountered in up to 10 patients.

We have analyzed 10,186 cancer genomes from 33 tumor types of the TCGA (The Cancer Genome Atlas^22^) and focused on the 143,444 frame shift mutations represented in this cohort (see Table S1). Translation of these mutations after re-annotation to a RefSeq annotation, starting in the protein reading frame, can lead to 70,439 unique peptides that are 10 or more amino acids in length (a cut-off we have set at a size sufficient to shape a distinct epitope in the context of MHC (Fig. 1a). The list of genes most commonly represented in the cohort and containing such frame shift mutations is headed nearly exclusively by tumor driver genes, such as NF1, RB, BRCA2 (Fig. 1b and Table S2) whose whole or partial loss of function apparently contributes to tumorigenesis. Note that a priori frame shift mutations are expected to result in loss of gene function more than a random SNV, and more independent of the precise position. In conclusion, NOPs initiated from a frameshift mutation and of a significant size are prevalent in tumors, and are enriched in cancer driver genes.

**Figure 2:**
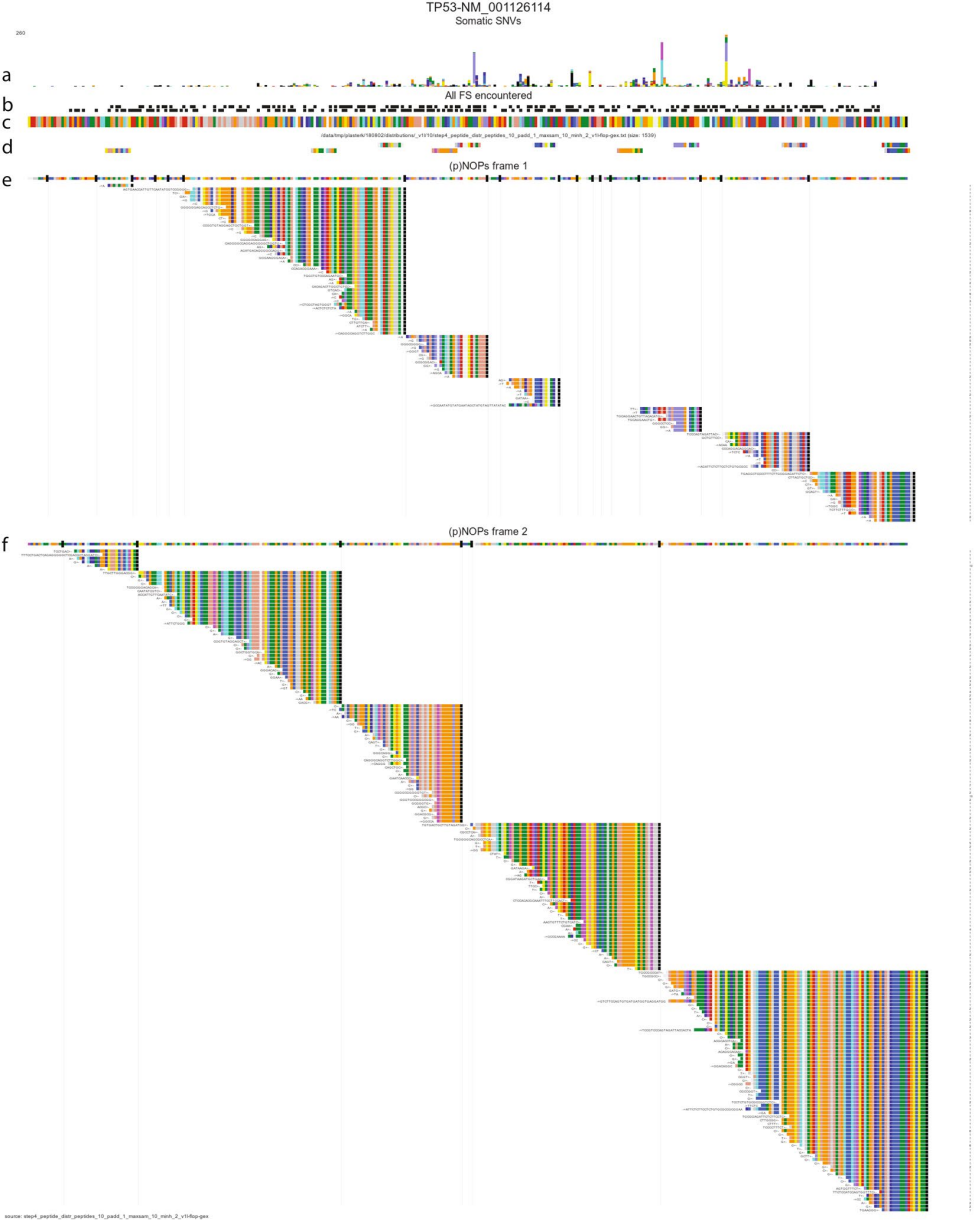
Neo open reading frame peptides (TCGA cohort) converge on common peptide sequences. Graphical representation in an isoform of TP53, where amino acids are colored distinctly. (**a**) somatic single nucleotide variants, (**b**). positions of frame shift mutations on the −1 and the +1 frame. (**c**) amino acid sequence of TP53. (**d**) Peptide (10aa) library (n = 1,000) selection. Peptides belonging to −1 or +1 frame are separated vertically (**e**,**f**) pNOPs for the different frames followed by all encountered frame shift mutations (rows), translated to a stop codon (lines) colored by amino acid.

Alignment of the translated NOP products onto the protein sequence reveals that a wide array of different frame shift mutations translate in a common downstream stretch of neo open reading frame peptides (‘NOPs’), as dictated by the −1 and +1 alternative reading frames. While we initially screened for NOPs of ten or more amino acids, their open reading frame in the out-of-frame genome often extends far beyond that search window. As a result we see (Fig. 2) that hundreds of different frame shift mutations all at different sites in the gene nevertheless converge on only a handful of NOPs. Similar patterns are found in other common driver genes (Supplementary Fig. S1).

Figure 2 illustrates that the precise location of a frame shift does not seem to matter much; the more or less straight slope of the series of mutations found in these 10,186 tumors indicates that it is not relevant for the biological effect (presumably reduction/loss of gene function) where the precise frame shift is, as long as translation stalls in the gene before the downstream remainder of the protein is expressed.

As can also be seen in Fig. 2, all frame shift mutations alter the reading frame to one of the two alternative frames. Therefore, for potential immunogenicity the relevant information is the sequence of the alternative ORFs and more precisely, the encoded peptide sequence between 2 stop codons. We term these peptides ‘proto Neo Open Reading Frame peptides’ or pNOPs, and generated a full list of all thus defined out of frame protein encoding regions in the human genome, of 10 amino acids or longer. We refer to the total sum of all Neo-ORFs as the Neo-ORFeome. The Neo-ORFeome contains all the peptide potential that the human genome can generate after simple frame-shift induced mutations. The size of the Neo-ORFeome is 25.6 Mb.

To investigate whether or not Nonsense Mediated Decay would wipe out frame shift mRNAs, we turned to a public repository containing read coverage for a large collection of cell lines (CCLE). We processed the data in a similar fashion as for the TCGA, identified the locations of frame shifts and subsequently found that, in line with the previous literature^23–25^, at least a large proportion of expressed genes also contained the frame shift mutation within the expressed mRNAs (Supplementary Fig. S2). On the mRNA level, NOPs can be detected in RNAseq data.

We next investigated how the number of patients relates to the number of NOPs. We sorted 10-mer peptides from NOPs by the number of new patients that contain the queried peptide. Assessed per tumor type, frame shift mutations in genes with very low to absent mRNA expression were removed to avoid overestimation. Of note NOP sequences are sometimes also encountered in the normal ORFeome, presumably as result of naturally occuring isoforms (e.g. Supplementary Fig. S3). Also these peptides were excluded.

**Figure 3:**
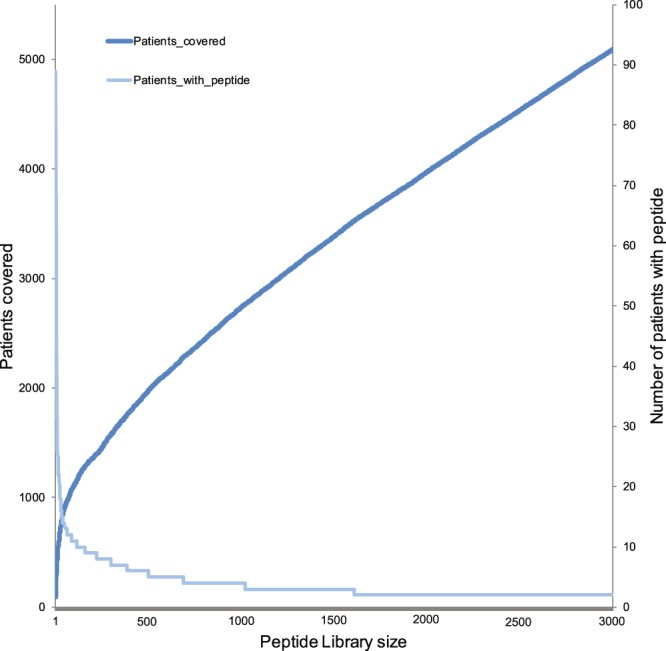
a recurrent peptide selection procedure can generate a ‘fixed’ library to cover up to 50% of the TCGA cohort. Graph depicts the number of unique patients accomodated by a a growing library of 10-mer peptides, picked in descending order of the number patients with that sequence in their NOPs. A peptide is only added if it adds a new patient from the cohort. Light shade depicts the number of patients containing the peptide that was included.

Taking into account the rules described above, and demanding that the addition of a peptide adds at least 1 new patient, we can create a library of possible ‘vaccines’ that is optimally geared towards covering the TCGA cohort, a cohort large enough that, also looking at the data presented here, it is representative of future patients (Table S3). Using this strategy 30% of all patients can be covered with a fixed collection of only 1,244 peptides of length 10 (Fig. 3). Since tumors will regularly have more than 1 frame shift mutation, one can use a ‘cocktail’ of different NOPs to optimally attack a tumor. Indeed, given a library of 1,244 peptides, 27% of the covered TCGA patients contain 2 or more ‘vaccine’ candidates. We ran the pNOPs through the NetMHC4.0 algorithm to predict MHC class 1 binding for HLA-A0201 (Table S3). 56% of the library pNOPs (with frame shift covering 2 or more TCGA patients) contain sequences that are considered to have weak to strong binding properties. This is highly enriched compared to all pNOPs (33%), where no or one TCGA frameshift mutation has been encountered (Fisher’s Exact test p < 2.2 10^-16^). In conclusion, using a limited pool with optimal patient inclusion of vaccines, a large proportion of patients is covered.

**Figure 4:**
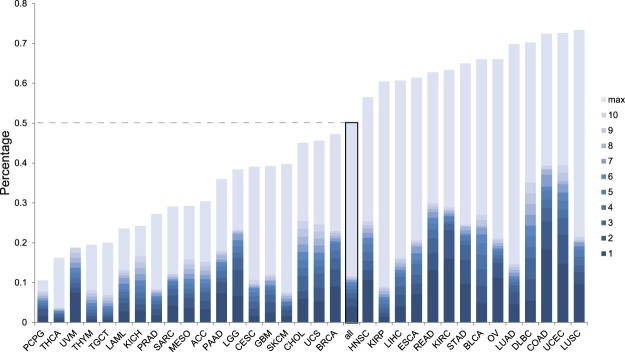
For some cancers up to 70% of patients contain a recurrent NOP. TCGA cohort ratio of patients separated by tumor type that could be ‘helped’ using optimally selected peptides for genes encountered most often within a cancer. Coloring represents the ratio, using 1, 2. 10 genes, or using all encountered genes (lightest shade).

Strikingly, using only 6 genes (TP53, ARID1A, KMT2D, GATA3, APC, PTEN), already 10% of the complete TCGA cohort is covered (Supplement Table S4). Separating this by the various tumor types, we find that for some cancers (like Pheochromocytoma and Paraganglioma (PCPG) or Thyroid carcinoma (THCA)) the hit rate is low, while for others up to 39% can be covered even with only 10 genes (Colon adenocarcinoma (COAD) using 60 peptides, Uterine Corpus Endometrial Carcinoma (UCEC) using 90 peptides), Fig. 4 and Table S4. At saturation (using all peptides encountered more than once) 50% of TCGA is covered and more than 70% can be achieved for specific cancer types (COAD, UCEC, Lung squamous cell carcinoma (LUSC) 72%, 73%, 73% respectively). As could be expected, these roughly follow the mutational load in the respective cancer types (Table S1). In addition some frame shifted genes are highly enriched in specific tumor types (e.g. VHL, GATA3. Supplementary Fig. S4). We conclude that at saturating peptide coverage, using only very limited set of genes, a large cohort of patients can be provided with off the shelf vaccines.

To validate the presence of NOPs, we used the targeted sequencing data on 10,129 patients from the MSK-IMPACT cohort^26^. For the 341–410 genes assessed in this cohort, we obtained strikingly similar results in terms of genes frequently affected by frame shifts and the NOPs that they create (Supplementary Fig. S5). Even within this limited set of genes, 86% of the library peptides (in genes targeted by MSK-IMPACT) were encountered in the patient set.

Since some cancers, like glioblastoma or pancreatic cancer, show survival expectancies after diagnosis measured in months rather than years (e.g. see^27^), it is of crucial importance to move as much of the work load and time line to the moment before diagnosis. Since the time of whole exome sequencing after biopsy is currently technically days, and since the scan of a resulting sequence against a public database describing these NOPs takes seconds, and the shipment of a peptide of choice days, a vaccination can be done theoretically within days and practically within a few weeks after biopsy. This makes it attractive to generate a stored and quality controlled peptide vaccine library based on the data presented here, possibly with replicates stored on several locations in the world. The synthesis in advance will - by economics of scale - reduce costs, allow for proper regulatory oversight, and can be quality certified, in addition to saving the patient time and thus provide chances. Since the vaccine repertoir is so limited, there is potential for a steep ‘learning curve’ to recognize the best vaccines. This approach will not replace other therapies, but be an additional option in the treatment repertoire. These advantages of scale also apply to other means of vaccination against these common neoantigens, by RNA- or DNA–based approaches (e.g.^28^), or recombinant bacteria (e.g.^29^).

It has not escaped our notice that the recognition of common antigens (resulting from different mutations) also suggests an approach for neoantigen application of the powerful CAR-T therapy (For recent review see^30^, and references therein), where the T-cells are directed not against a cell-type specific antigens (such as CD19 or CD20), but against a tumor specific neoantigen. E.g. once one functional T-cell against any of the common p53 NOPs (Fig. 2) is identified, the recognition domains can be engineered into T-cells for any future patient with such a NOP, and the constructs could similarly be deposited in an off-the-shelf library.

In this work, we have identified that various frame shift mutations can result in a source for common neo open reading frame peptides, suitable as pre-synthesized vaccines. This discovery, potentially combined with checkpoint inhibition, holds promise for future treatment strategies to help instruct our own immune system to defeat cancer.

## Methods

### TCGA frameshift mutations

Frame shift mutations were retrieved from Varscan and mutect files per tumor type via https://portal.gdc.cancer.gov/. Frame shift mutation contained within these files were extracted using custom perl scripts and used for the further processing steps using HG38 as reference genome build.

### CCLE frameshift mutations

For the CCLE cell line cohort, somatic mutations were retrieved from http://www.broadinstitute.org/ccle/data/browseData?conversationPropagation=begin (CCLE_hybrid_capture1650_hg19_NoCommonSNPs_NoNeutralVariants_CDS_2012.02.20.maf). Frame shift mutations were extracted using custom perl scripts using hg19 as reference genome.

### Refseq annotation

To have full control over the sequences used within our analyses, we downloaded the reference sequences from the NCBI website (2018-02-27) and extracted mRNA and coding sequences from the gbff files using custom perl scripts. Subsequently, mRNA and every exon defined within the mRNA sequences were aligned to the genome (hg19 and hg38) using the BLAT suite. The best mapping locations from the psl files were subsequently used to place every mRNA on the genome, using the separate exons to perform fine placement of the exonic borders. Using this procedure we also keep track of the offsets to enable placement of the amino acid sequences onto the genome.

### Mapping genome coordinate onto Refseq

To assess the effect of every mentioned frame shift mutation within the cohorts (CCLE or TCGA), we used the genome coordinates of the frameshifts to obtain the exact protein position on our reference sequence database, which were aligned to the genome builds. This step was performed using custom perl scripts taking into account the codon offsets and strand orientation, necessary for the translation step described below.

### Translation of FS peptides

Using the reference sequence annotation and the positions on the genome where a frame shift mutation was identified, the frame shift mutations were used to translate peptides until a stop codon was encountered. The NOP sequences were recorded and used in downstream analyses as described in the text.

### Verification of FS mRNA expression in the CCLE colorectal cancer cell lines

For a set of 59 colorectal cancer cell lines, the HG19 mapped bam files were downloaded from https://portal.gdc.cancer.gov/. Furthermore, the locations of FS mutations were retrieved from CCLE_hybrid_capture1650_hg19_NoCommonSNPs_NoNeutralVariants_CDS_2012.02.20.maf (http://www.broadinstitute.org/ccle/data/browseData?conversationPropagation=begin), by selection only frameshift entries. Entries were processed similarly to to the TCGA data, but this time based on a HG19 reference genome. To get a rough indication that a particular location in the genome indeed contains an indel in the RNAseq data, we first extracted the count at the location of a frameshift by making use of the pileup function in samtools. Next we used the special tag XO:1 to isolate reads that contain an indel in it. On those bam files we again used the pileup function to count the number of reads containing an indel (assuming that the indel would primarily be found at the frameshift instructed location). Comparison of those 2 values can then be interpreted as a percentage of indel at that particular location. To reduce spurious results, at least 10 reads needed to be detected at the FS location in the original bam file.

### Defining peptide library

To define peptide libraries that are maximized on performance (covering as many patients with the least amount of peptides) we followed the following procedure. From the complete TCGA cohort, FS translated peptides of size 10 or more (up to the encountering of a stop codon) were cut to produce any possible 10-mer. Then in descending order of patients containing a 10-mer, a library was constructed. A new peptide was added only if an additional patient in the cohort was included. peptides were only considered if they were seen 2 or more times in the TCGA cohort, if they were not filtered for low expression (see Filtering for low expression section), and if the peptide was not encountered in the orfeome (see Filtering for peptide presence orfeome). In addition, since we expect frame shift mutations to occur randomly and be composed of a large array of events (insertions and deletions of any non triplet combination), frame shift mutations being encountered in more than 10 patients were omitted to avoid focusing on potential artefacts. Manual inspection indicated that these were cases with e.g. long stretches of Cs, where sequencing errors are common.

### Filtering for low expression

Frameshift mutations within genes that are not expressed are not likely to result in the expression of a peptide. To take this into account we calculated the average expression of all genes per TCGA entity and arbitrarily defined a cutoff of 2 log2 units as a minimal expression. Any frameshift mutation where the average expression within that particular entity was below the cutoff was excluded from the library. This strategy was followed, since mRNA gene expression data was not available for every TCGA sample that was represented in the sequencing data set. Expression data (RNASEQ v2) was pooled and downloaded from the R2 platform (http://r2.amc.nl). In current sequencing of new tumors with the goal of neoantigen identification such mRNA expression studies are routine and allow routine verification of presence of mutant alleles in the mRNA pool.

### Filtering for peptide presence orfeome

Since for a small percentage of genes, different isoforms can actually make use of the shifted reading frame, or by chance a 10-mer could be present in any other gene, we verified the absence of any picked peptide from peptides that can be defined in any entry of the reference sequence collection, once converted to a collection of tiled 10-mers.

### Generation of cohort coverage by all peptides per gene

To generate overviews of the proportion of patients harboring exhaustive FS peptides starting from the most mentioned gene, we first pooled all peptides of size 10 by gene and recorded the largest group of patients per tumor entity. Subsequently we picked peptides identified in the largest set of patients and kept on adding a new peptide in descending order, but only when at least 1 new patient was added. Once all patients containing a peptide in the first gene was covered, we progressed to the next gene and repeated the procedure until no patient with FS mutations leading to a peptide of size 10 was left.

### proto-NOP (pNOP) and Neo-ORFeome

proto NOPs are those peptide products that result from the translation of the gene products when the reading frame is shifted by −1 or +1 base (so out of frame). Collectively, these pNOPs form the Neo-Orfeome.As such we generated a pNOP reference base of any peptide with length of 10 or more amino acids, from the RefSeq collection of sequences. Two notes: the minimal length of 10 amino acids is a choice; if one were to set the minimal window at 8 amino acids the total numbers go up a bit, e.g. the 30% patient covery of the library goes up. On a second note: we limited our definition to ORFs that can become in frame after a single insertion deletion on that location; this includes obviously also longer insertion or deletion stretches than +1 or −1. The definition has not taken account more complex events that get an out-of-frame ORF in frame, such as mutations creating or deleting splice sites, or a combination of two frame shifts at different sites that result in bypass of a natural stop codon; these events may and will occur, but counting those in will make the definition of the Neo-ORFeome less well defined. For the magnitude of the numbers these rare events do not matter much.

### Visualizing nops

Visualization of the nops was performed using custom perl scripts, which were assembled such that they can accept all the necessary input data structures such as protein sequence, frameshited protein sequences, somatic mutation data, library definitions, and the peptide products from frameshift translations.

### MHC class I binding prediction

To assess the MHC class I binding potential, NetMHC4.0 was utilized on every 10-mer that can be defined from a sliding window, with a stepsize of 1 amino acid (on the complete Neo ORFeome). NetMHC4.0 was executed in peptide mode (-p) with default settings (using HLA-A0201 as allele). Hits designated as weak binding (WB) and strong binding (SB) were kept and mapped back onto the pNOPs that contain these sequences. pNOPs that were selected in the library were flagged with MHC binding potential accordingly.

## Supplementary information

Figures S1-S5

Tables S1-S4

## Data Availability

The PERL scripts are available from the autors upon reasonable request. All the data used in this study can be downloaded from the GDC, Broad institute or the NCBI (see also the specific method sections).
