# Supplementary material for: A library of Neo Open Reading Frame peptides (NOPs) as a sustainable resource of common neoantigens in up to 50% of cancer patients
Source: Sci Rep. 2019 Apr 29;9:6577. doi: 10.1038/s41598-019-42729-2 (PMC6488612; doi:10.1038/s41598-019-42729-2)
Supplement: Supplementary file 1 — Figures S1-S5 [file 41598_2019_42729_MOESM1_ESM.pdf]

A library of Neo Open Reading Frame peptides (NOPs) as a sustainable resource of common neoantigens in up to 50% of cancer patients

Jan Koster and Ronald H.A. Plasterk

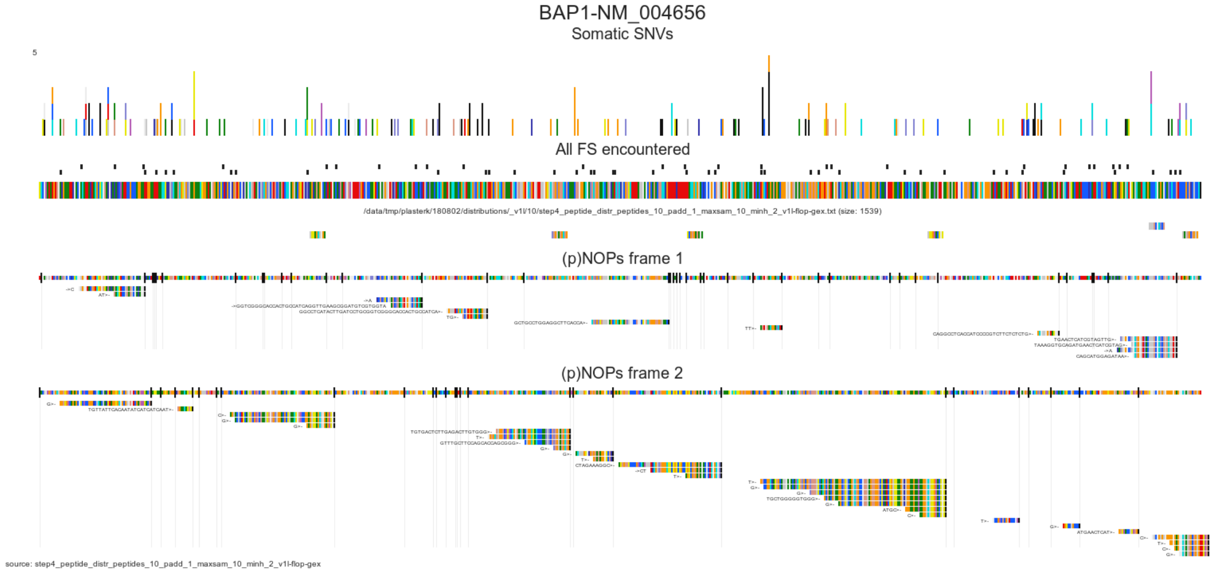

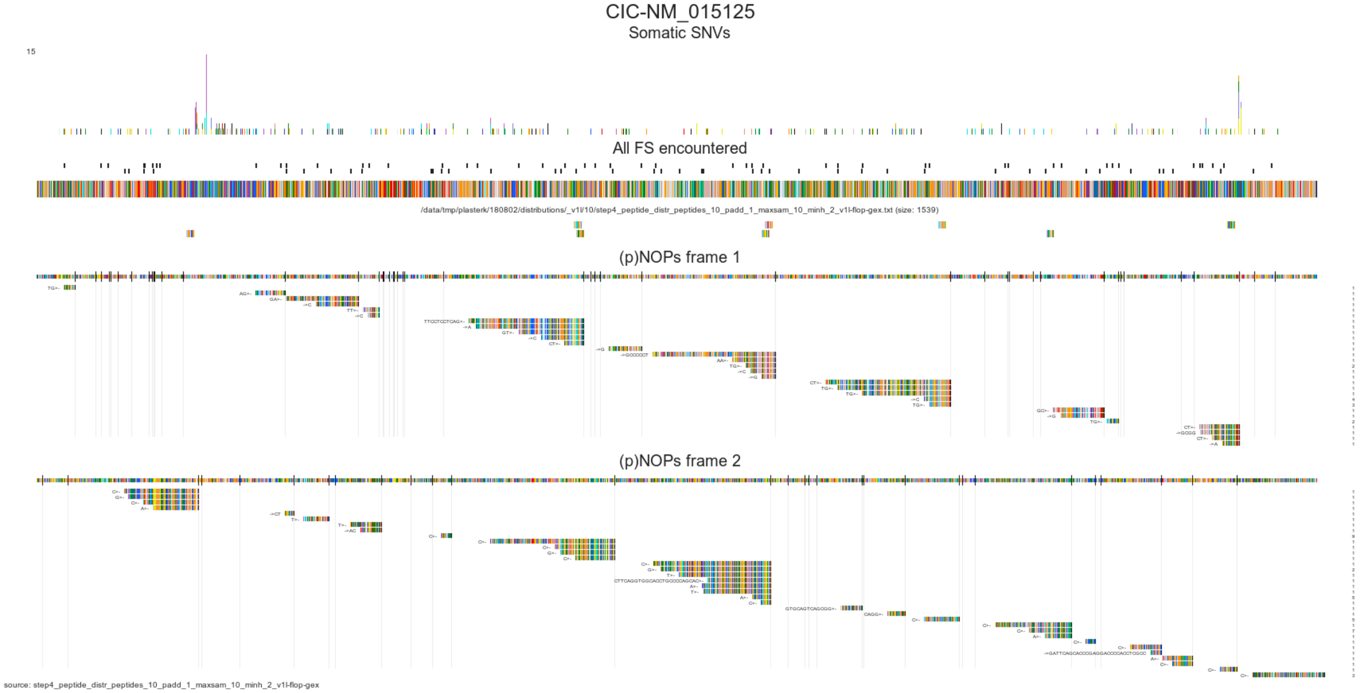

Supplemental Figure 1. Examples of NOPs
Small selection of genes containing NOPs of 10 or more amino acids

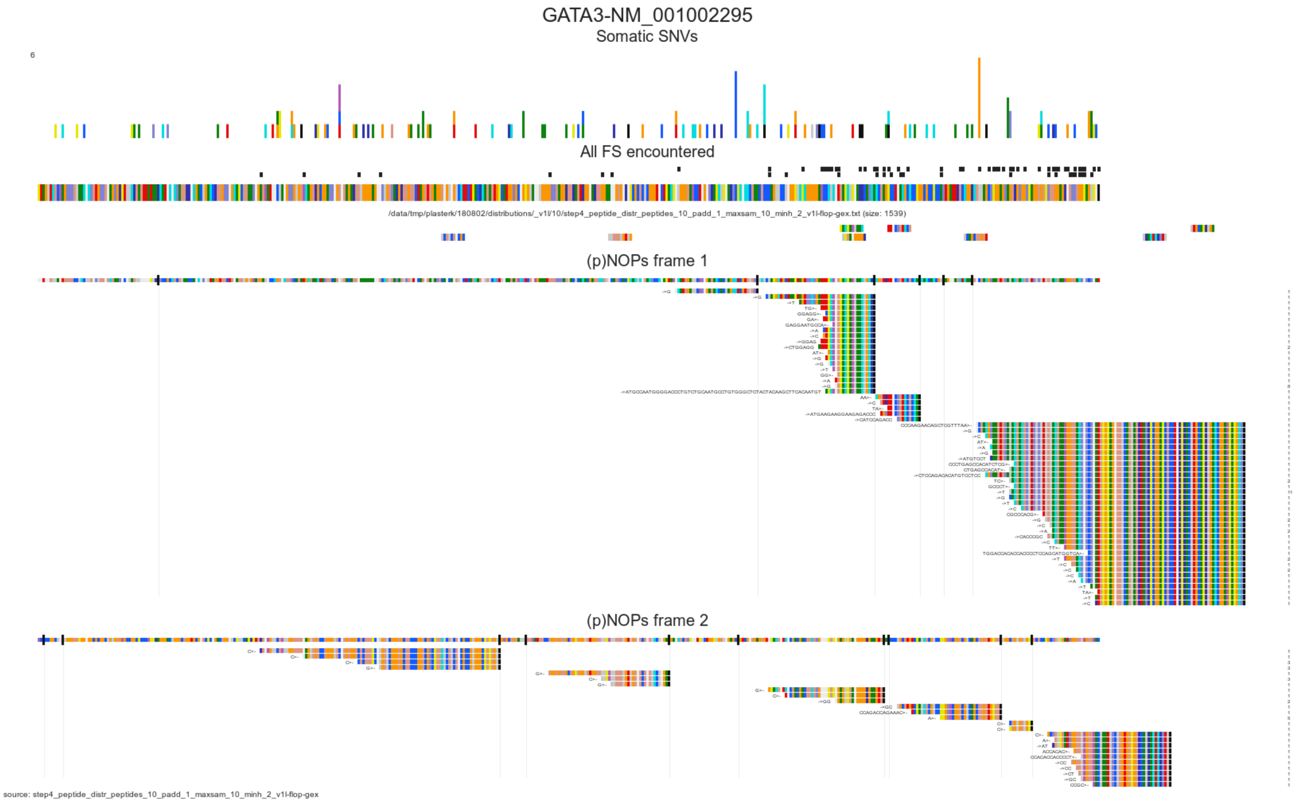
GATA3-NM_001002295
Somatic SNVs
6
All FS encountered
/data/tmp/plasterk/180802/distributions/_v1l/10/step4_peptide_distr_peptides_10_padd_1_maxsam_10_minh_2_v1l-flop-gex.txt (size: 1539)
(p)NOPs frame 1
(p)NOPs frame 2
source: step4_peptide_distr_peptides_10_padd_1_maxsam_10_minh_2_v1l-flop-gex

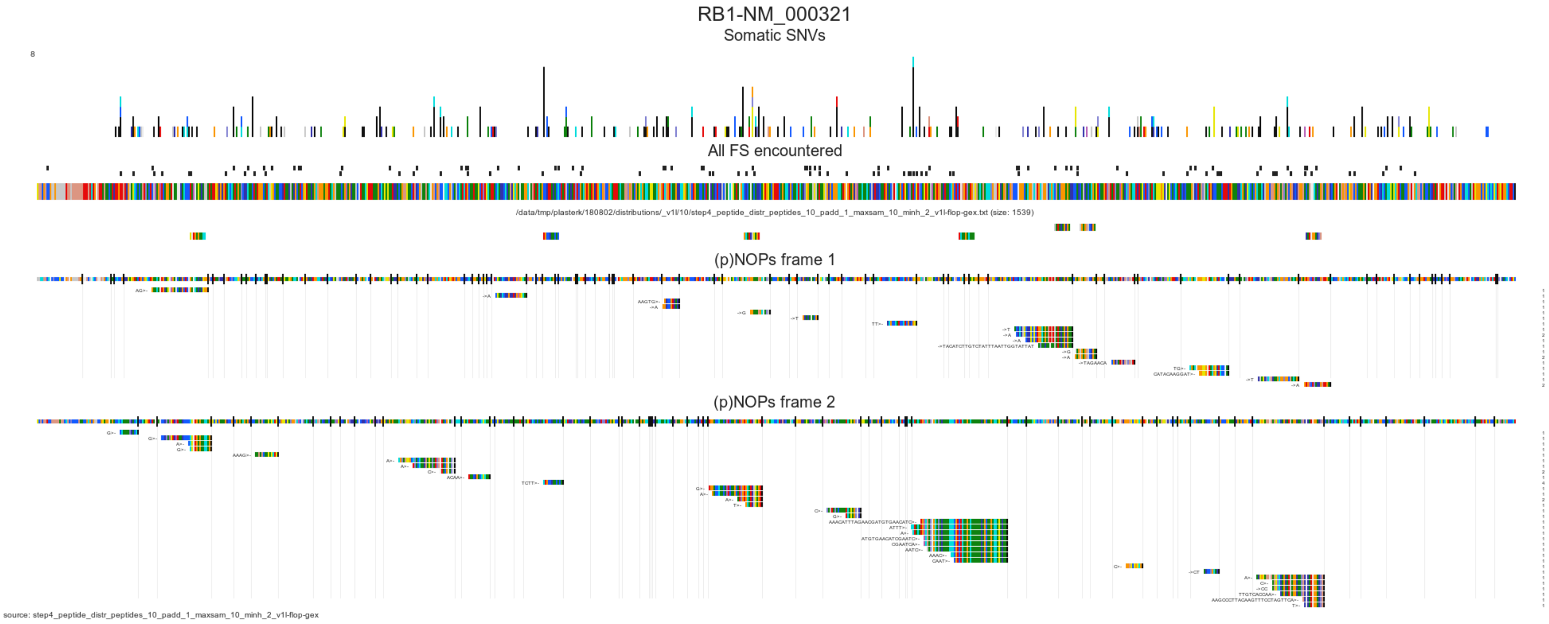
RB1-NM_000321
Somatic SNVs
8
All FS encountered
/data/tmp/plasterk/180802/distributions/_v1l/10/step4_peptide_distr_peptides_10_padd_1_maxsam_10_minh_2_v1l-flop-gex.txt (size: 1539)
(p)NOPs frame 1
(p)NOPs frame 2
source: step4_peptide_distr_peptides_10_padd_1_maxsam_10_minh_2_v1l-flop-gex

PIK3R1-NM_181524
Somatic SNVs

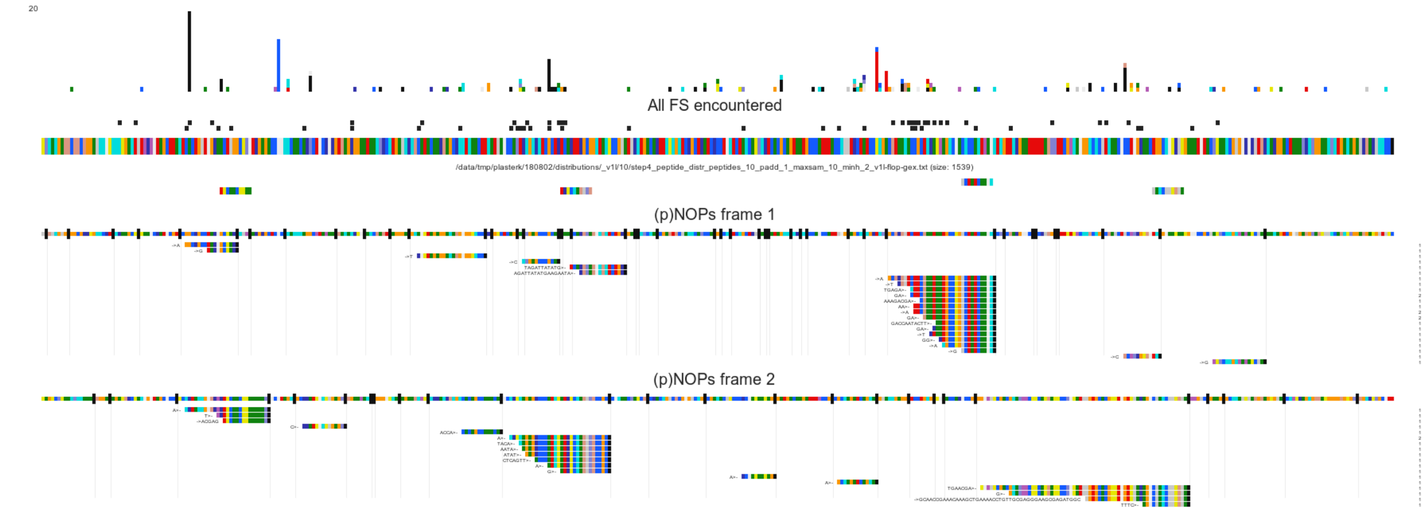

source: step4_peptide_distr_peptides_10_padd_1_maxsam_10_minh_2_v1l-flop-gex

PTEN-NM_000314
Somatic SNVs

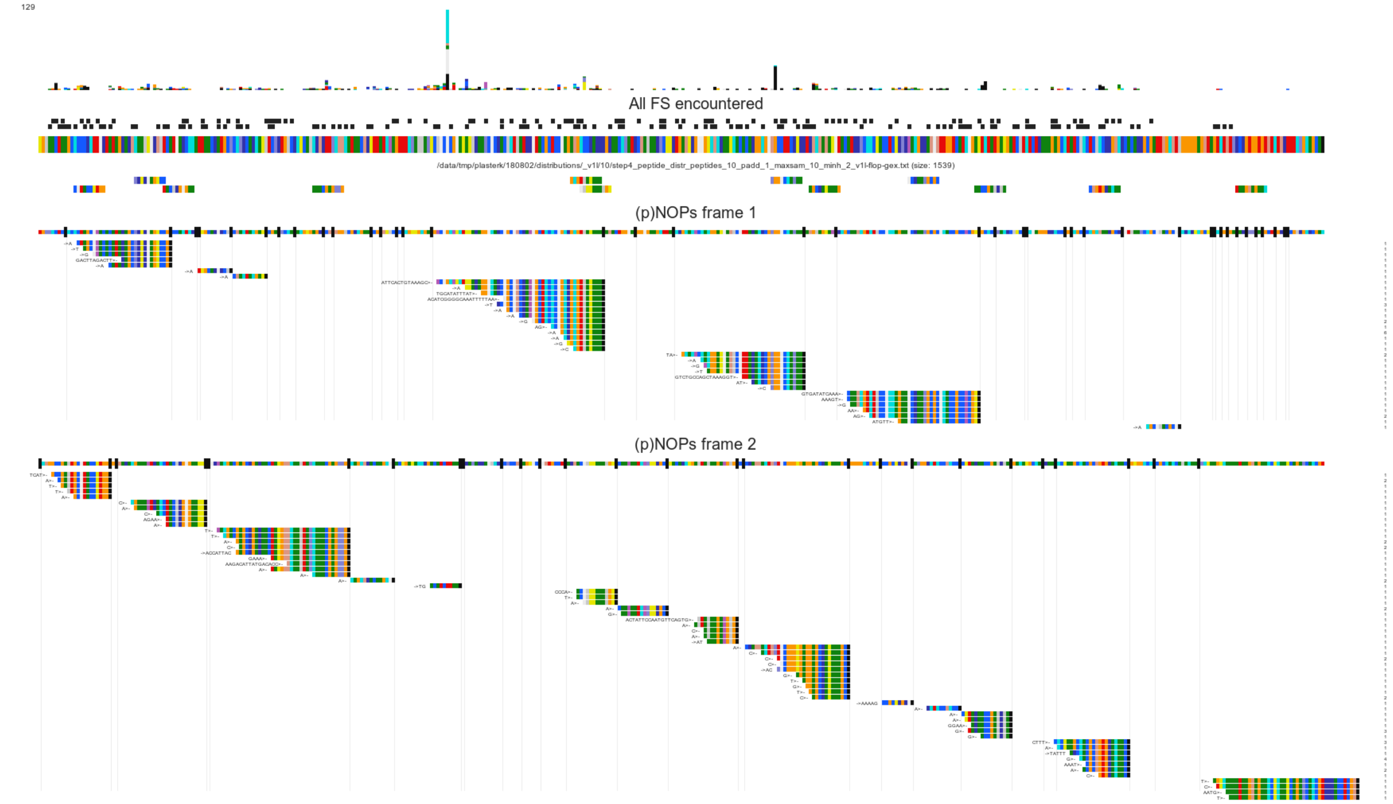

source: step4_peptide_distr_peptides_10_padd_1_maxsam_10_minh_2_v1l-flop-gex

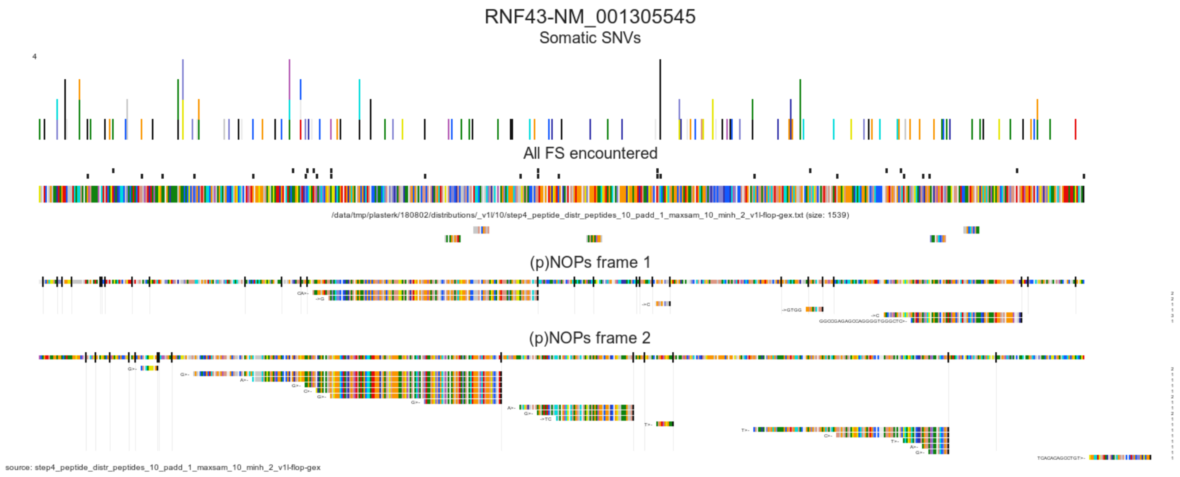
RNF43-NM_001305545
Somatic SNVs
All FS encountered
/data/tmp/plasterk/180802/distributions/_v1l/10/step4_peptide_distr_peptides_10_padd_1_maxsam_10_minh_2_v1l-flop-gex.txt (size: 1539)
(p)NOPs frame 1
(p)NOPs frame 2
source: step4_peptide_distr_peptides_10_padd_1_maxsam_10_minh_2_v1l-flop-gex

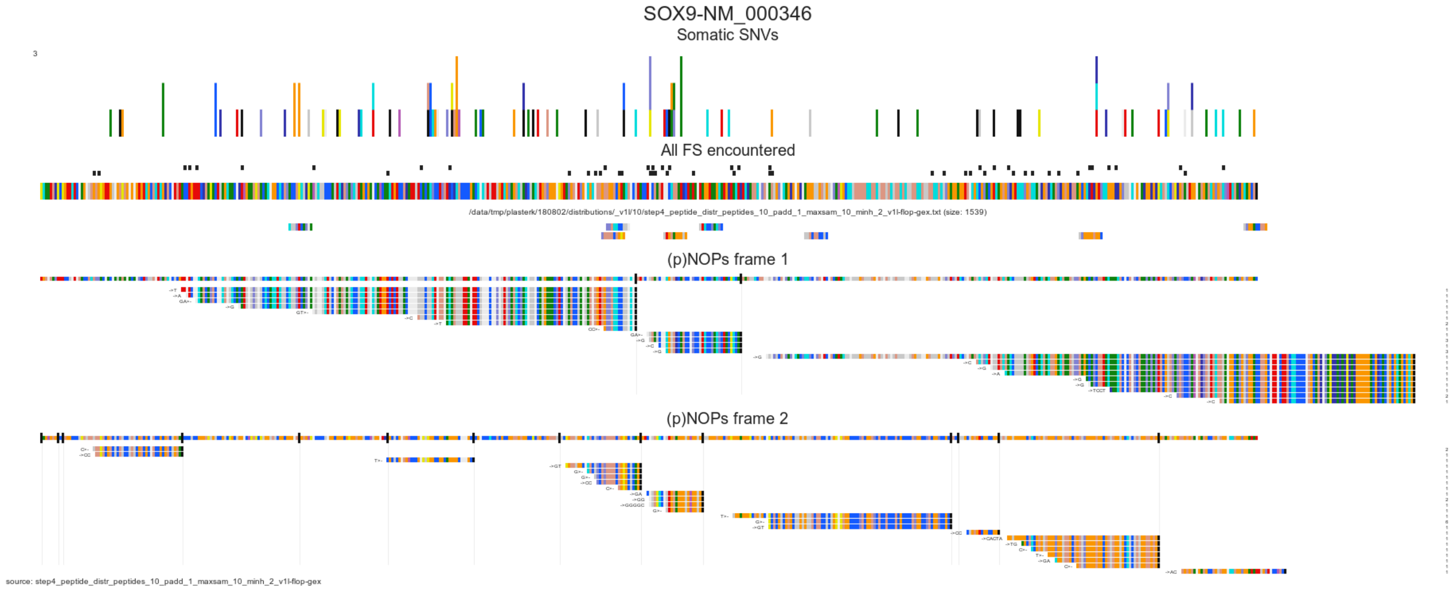
SOX9-NM_000346
Somatic SNVs
All FS encountered
/data/tmp/plasterk/180802/distributions/_v1l/10/step4_peptide_distr_peptides_10_padd_1_maxsam_10_minh_2_v1l-flop-gex.txt (size: 1539)
(p)NOPs frame 1
(p)NOPs frame 2
source: step4_peptide_distr_peptides_10_padd_1_maxsam_10_minh_2_v1l-flop-gex

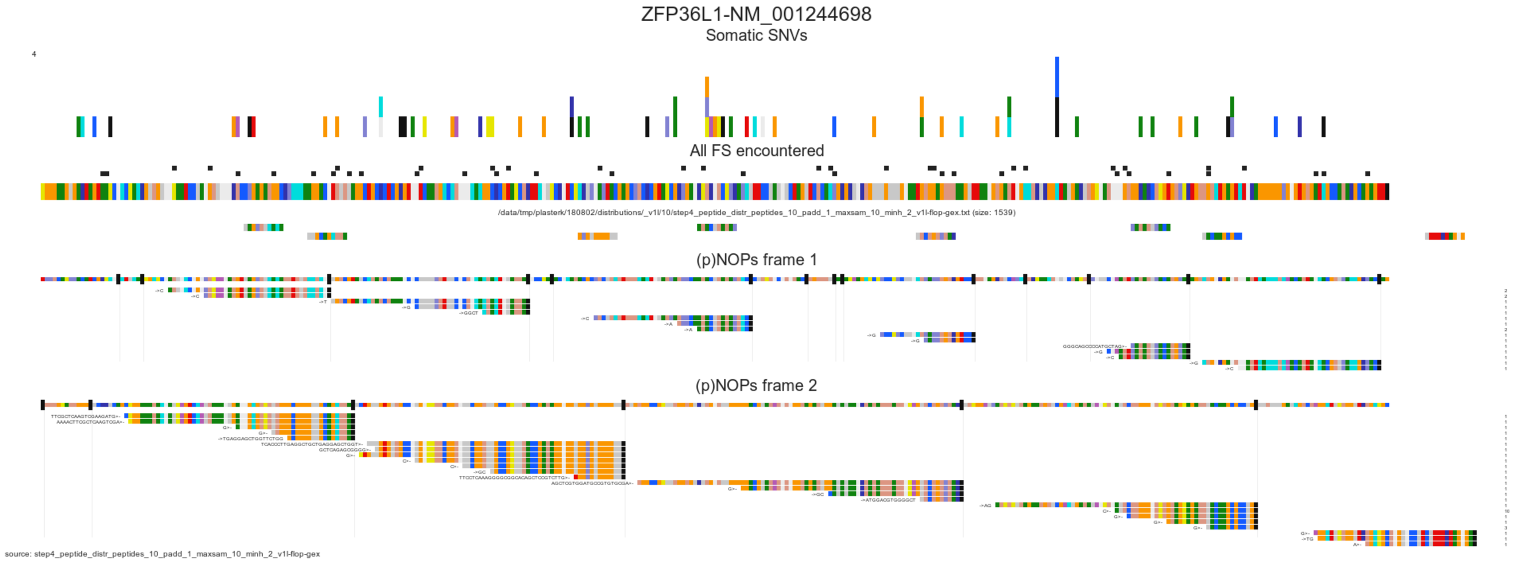
ZFP36L1-NM_001244698
Somatic SNVs
All FS encountered
/data/tmp/plasterk/180802/distributions/_v1l/10/step4_peptide_distr_peptides_10_padd_1_maxsam_10_minh_2_v1l-flop-gex.txt (size: 1539)
(p)NOPs frame 1
(p)NOPs frame 2
source: step4_peptide_distr_peptides_10_padd_1_maxsam_10_minh_2_v1l-flop-gex

VHL-NM_000551

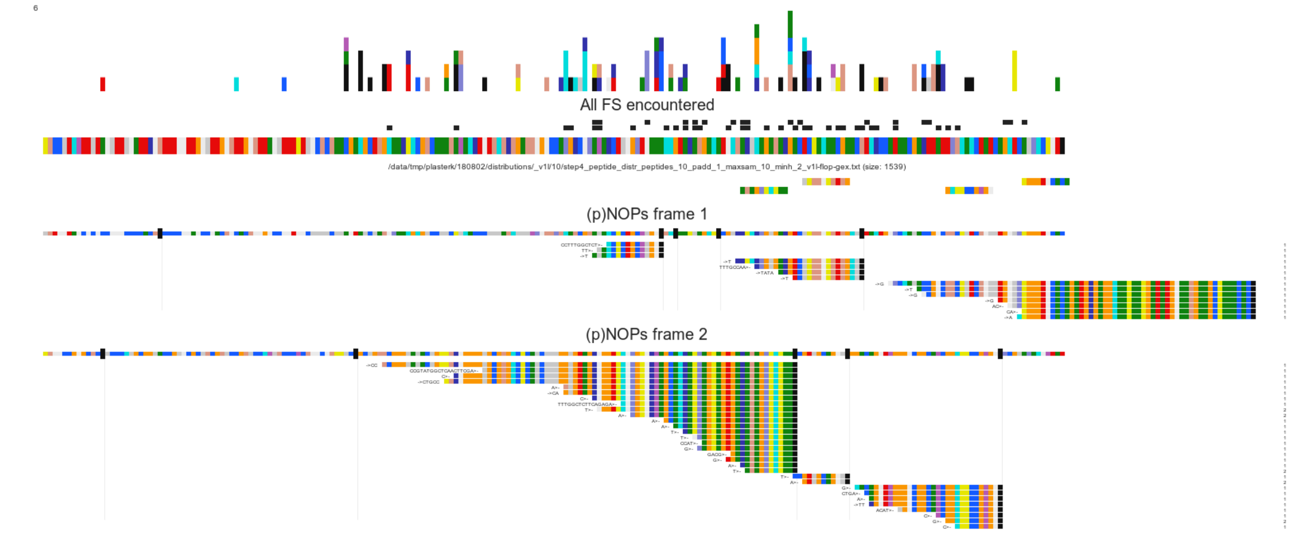

source: step4_peptide_distr_peptides_10_padd_1_maxsam_10_minh_2_v1l-flop-gex

ATRX-NM_138270

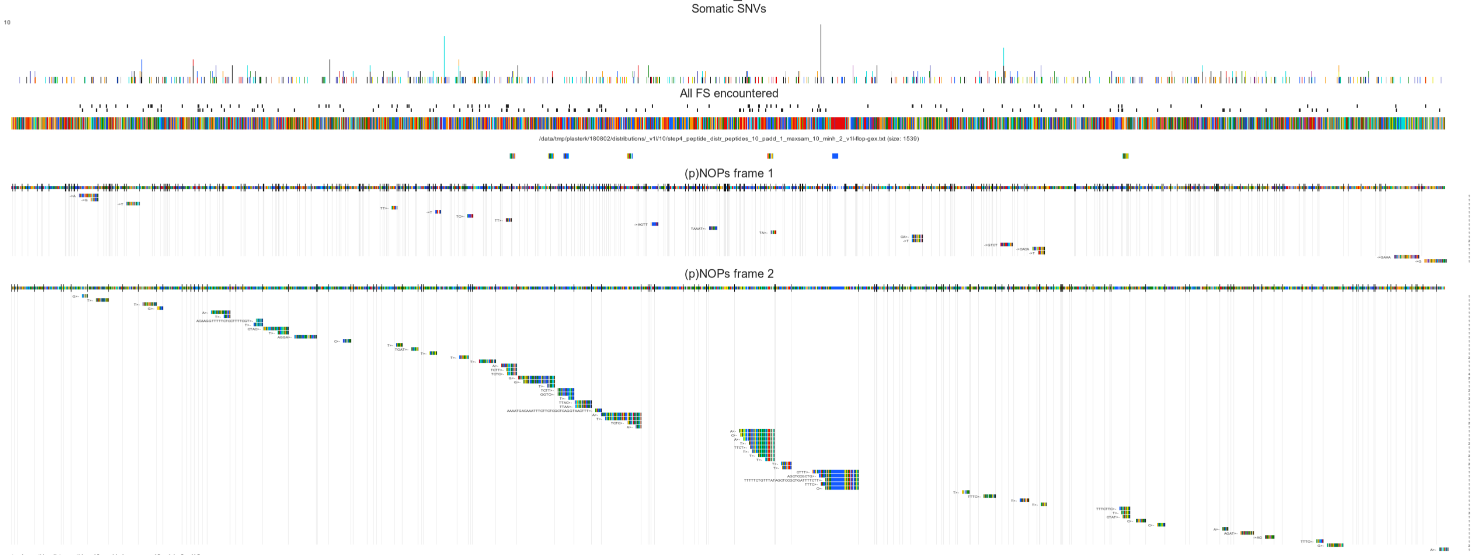

source: step4_peptide_distr_peptides_10_padd_1_maxsam_10_minh_2_v1l-flop-gex

a

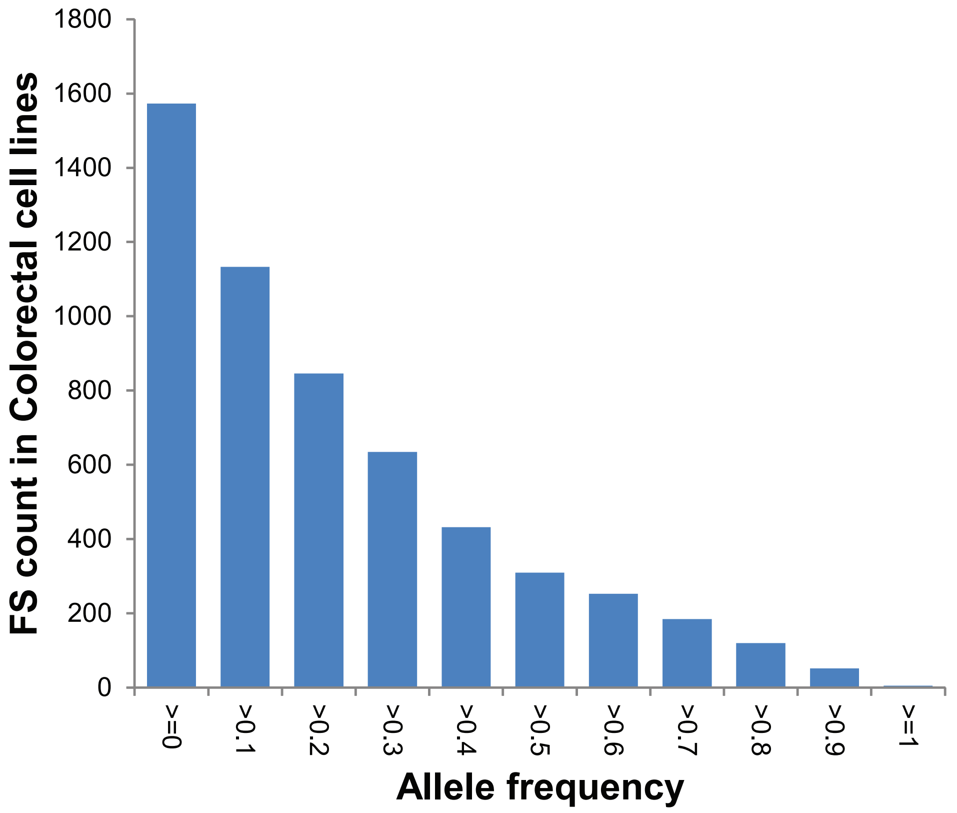

b

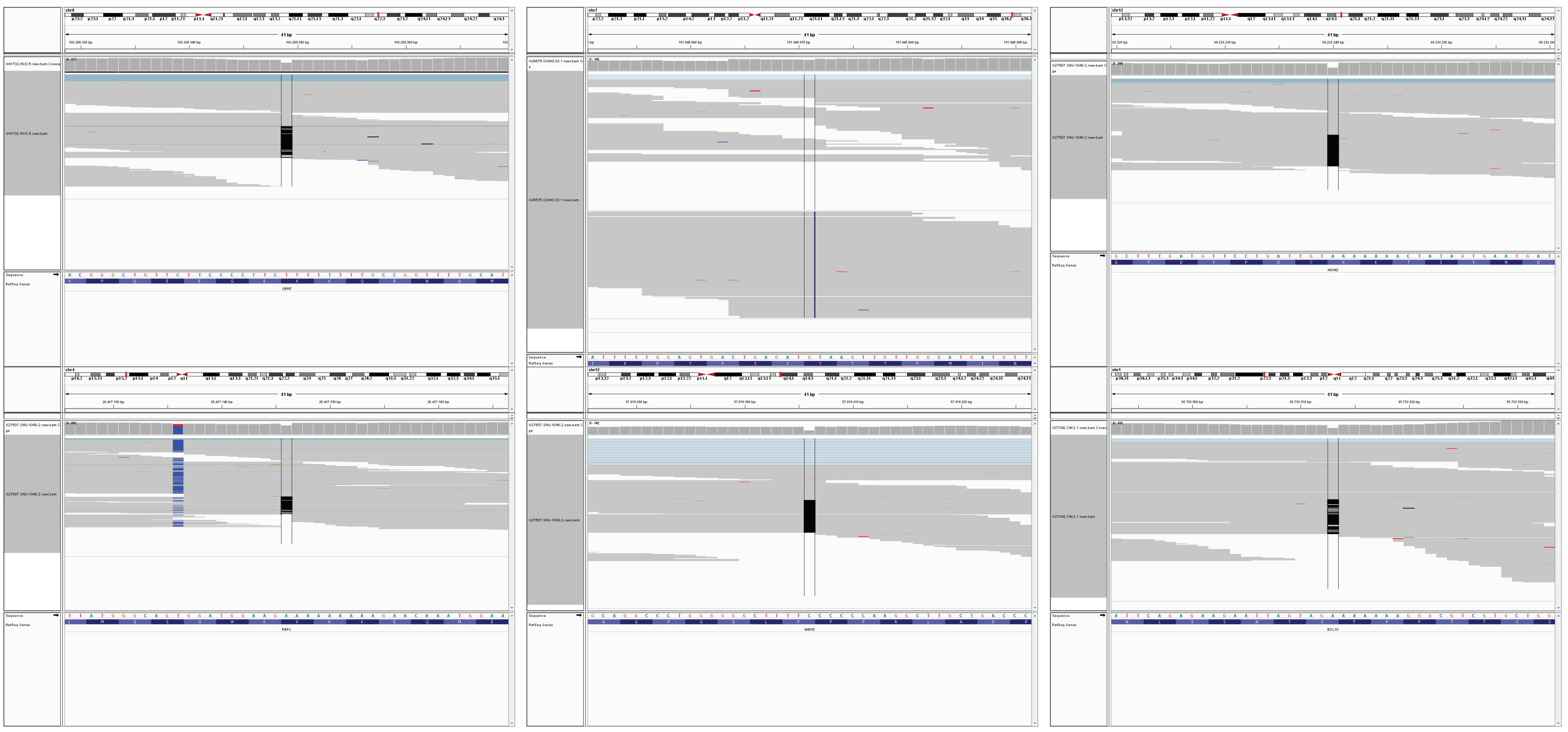

Supplemental Figure 2. Frame shift presence in mRNA from 58 CCLE colorectal cancer cell lines

a. Cumulative counting of RNAseq allele frequency (Samtools mpileup (XO:1/all)) at the genomic position of DNA detected frame shift mutations.

b. IGV examples of frame shift mutations in the BAM files of CCLE cell lines

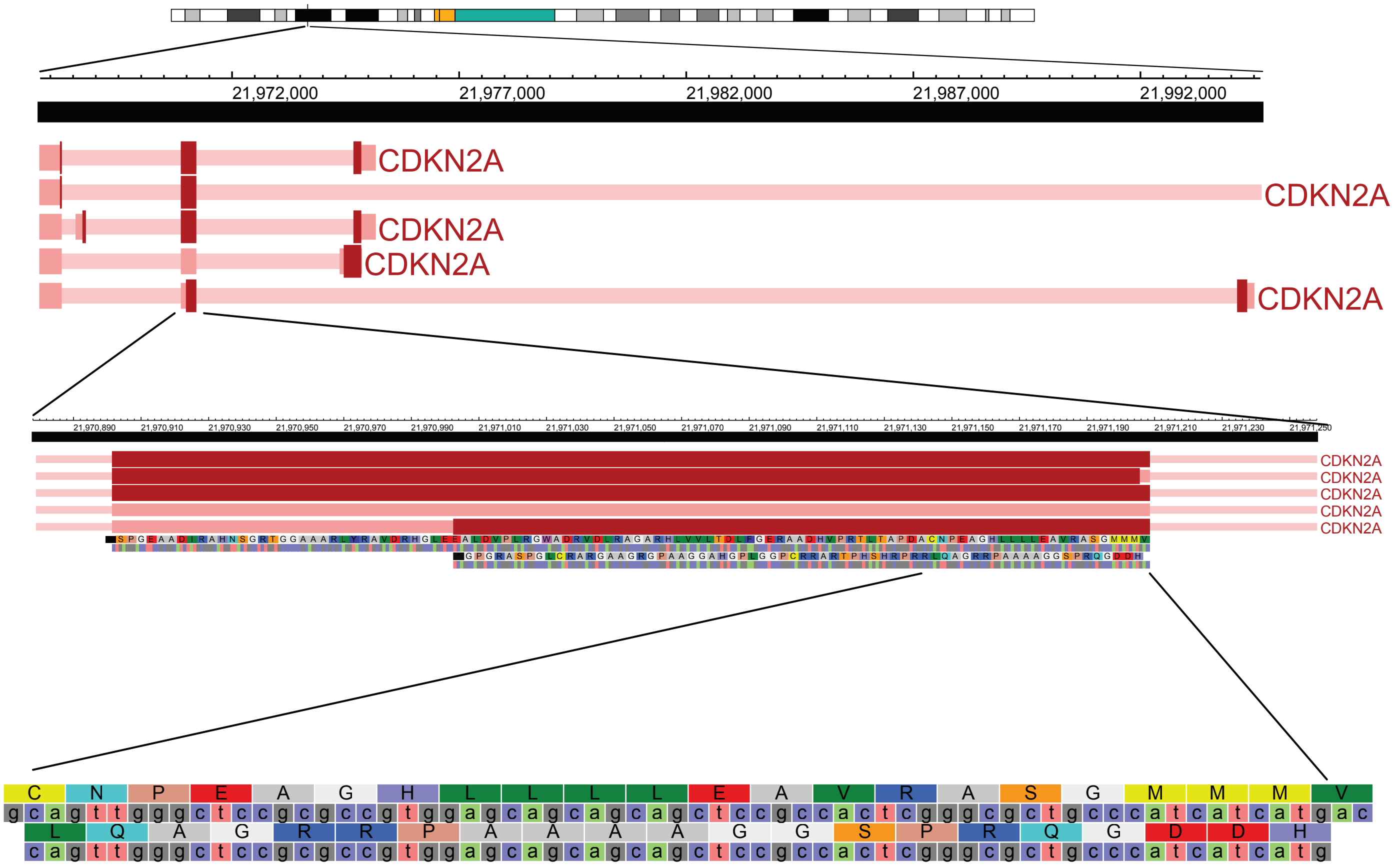

Supplemental Figure 3. Example of normal isoforms, using shifted frame

Genome model of CDKN2A with the different isoforms are shown on the minus strand of the genome. Zoom of the middle exon depicts the 2 reading frames that are encountered in the different isoforms.

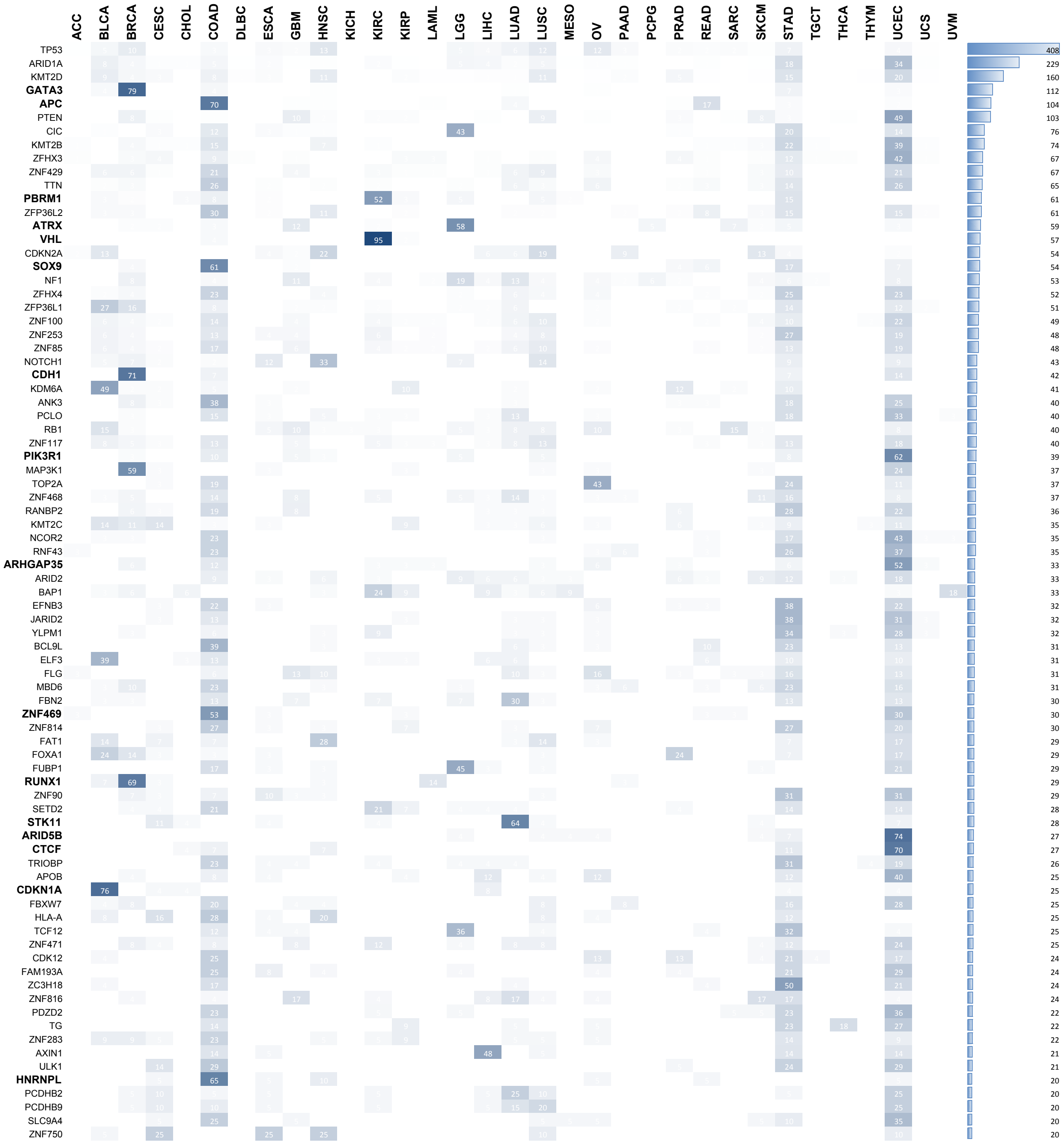

Supplemental Figure 4. Gene prevalence vs Cancer type

Percentage of frameshift mutations (resulting in peptides of 10 aa or longer), assessed by the type of cancer in the TCGA cohort. Genes where 50% or more of the frameshifts occur within a single tumor type are indicated in bold.

a

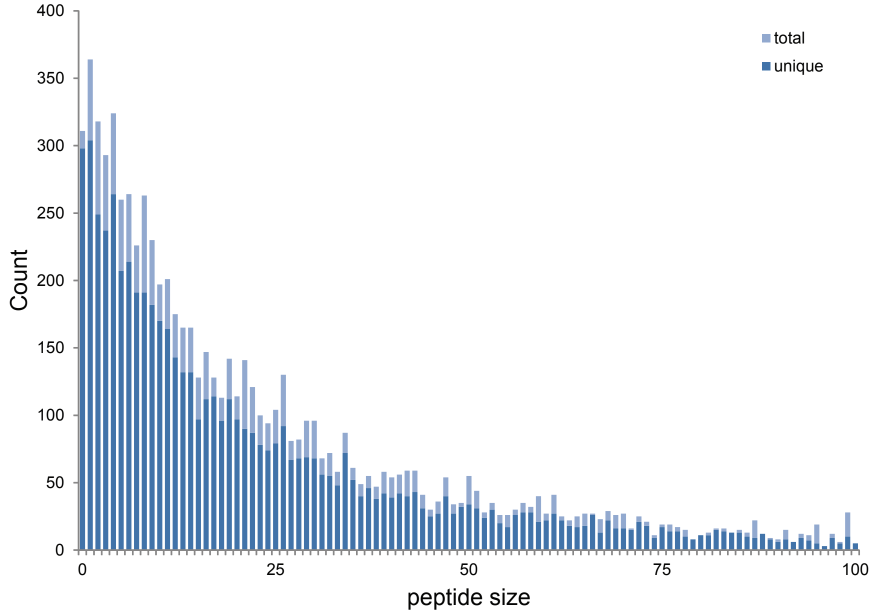
400
350
300
250
200
150
100
50
0
Count
0
25
50
75
100
peptide size
total
unique

b

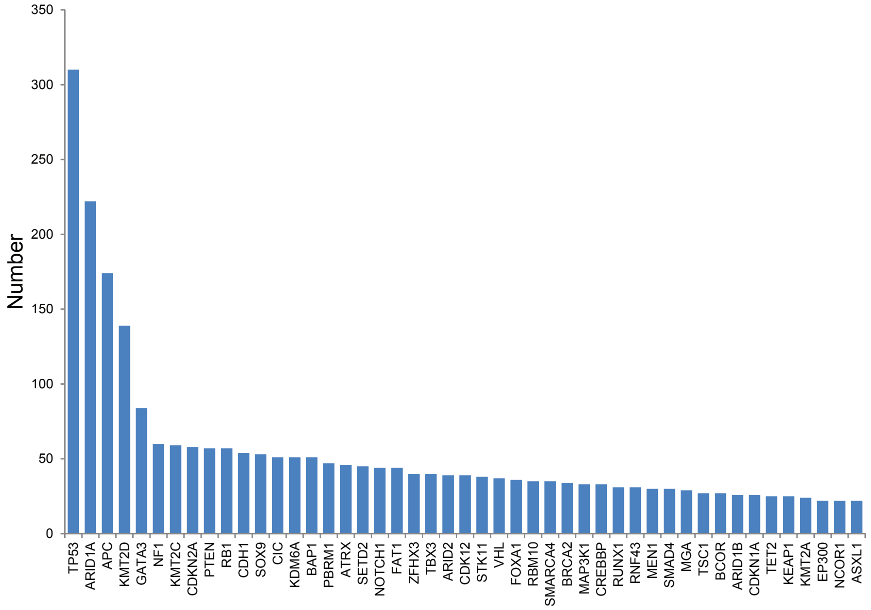
350
300
250
200
150
100
50
0
Number
TP53
ARID1A
APC
KMT2D
GATA3
NF1
KMT2C
CDKN2A
PTEN
RB1
CDH1
SOX9
CIC
KDM6A
BAP1
PBRM1
ATRX
SETD2
NOTCH1
FAT1
ZFHX3
TBX3
ARID2
CDK12
STK11
VHL
FOXA1
RBM10
SMARCA4
BRCA2
MAP3K1
CREBBP
RUNX1
RNF43
MEN1
SMAD4
MGA
TSC1
BCOR
ARID1B
CDKN1A
TET2
KEAP1
KMT2A
EP300
NCOR1
ASXL1

c

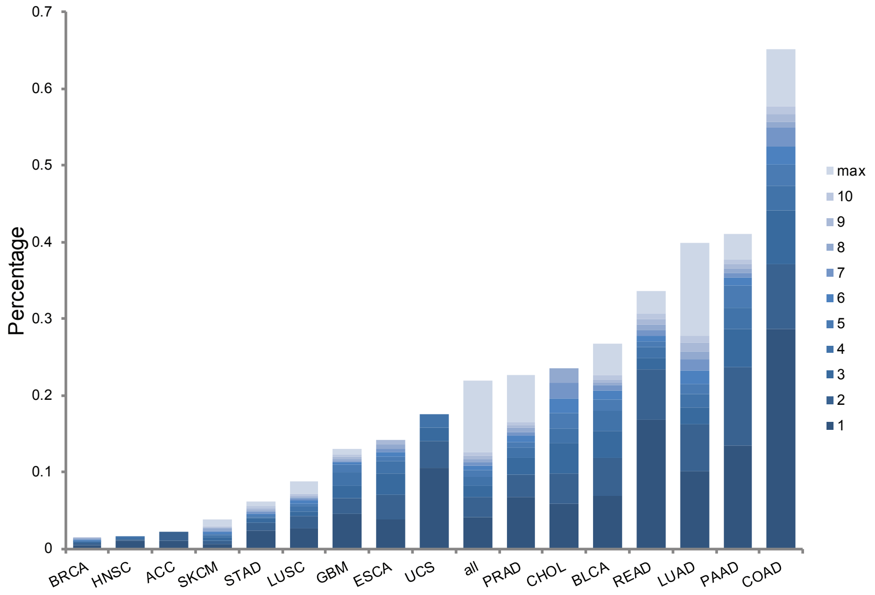
0.7
0.6
0.5
0.4
0.3
0.2
0.1
0
Percentage
BRCA
HNSC
ACC
SKCM
STAD
LUSC
GBM
ESCA
UCS
all
PRAD
CHOL
BLCA
READ
LUAD
PAAD
COAD
max
10
9
8
7
6
5
4
3
2
1

d

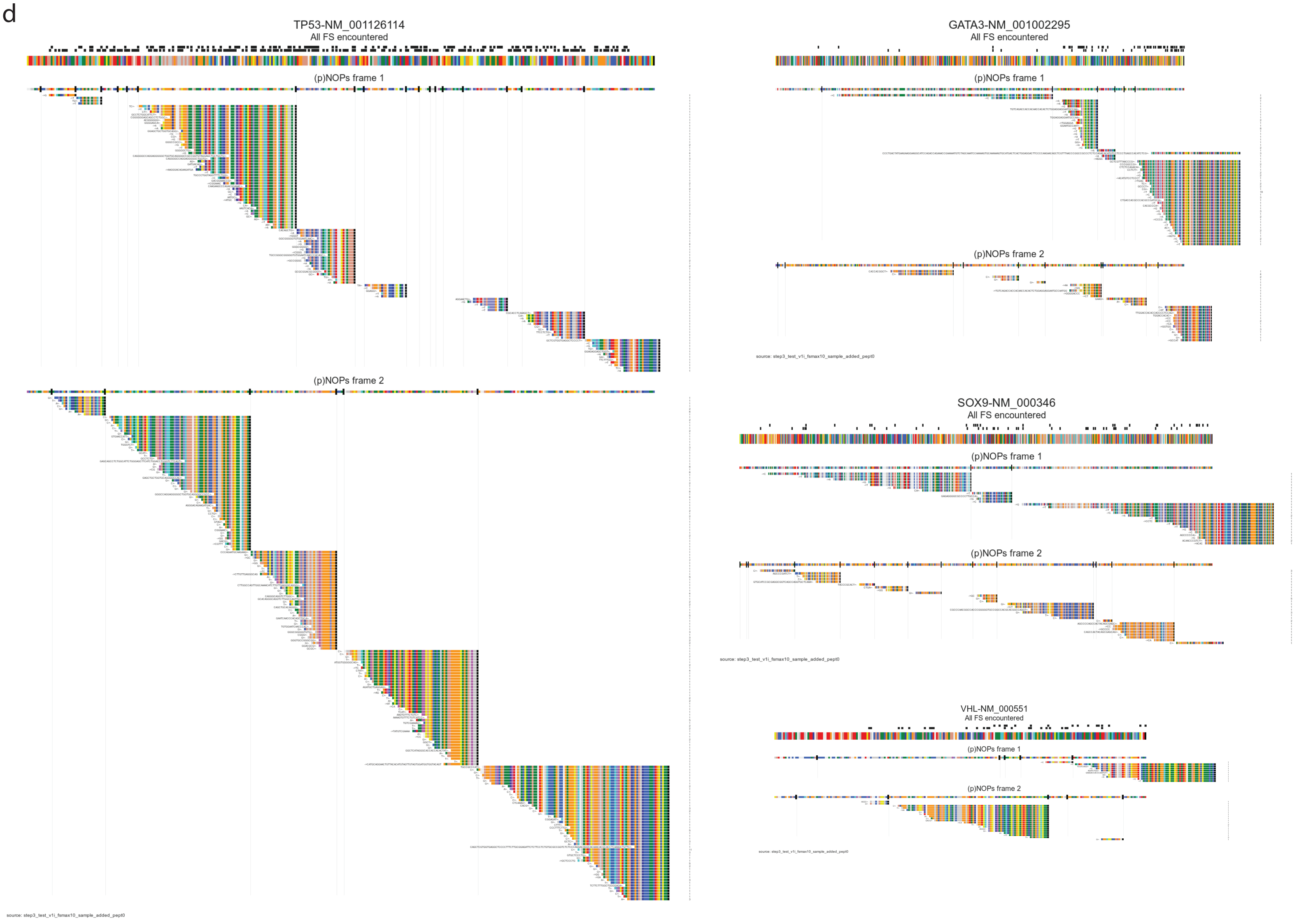

Supplemental Figure 5. NOPs in the MSK-IMPACT study

Frame shift analysis in the targeted sequencing panel of the MSK-IMPACT study, covering up to 410 genes in more 10,129 patients (with at least 1 somatic mutation). a. FS peptide length distribution, b. Gene count of patients containing NOPs of 10 or more amino acids. c. Ratio of patients separated by tumor type that possess a neo epitope using optimally selected peptides for genes encountered most often within a cancer. Coloring represents the ratio, using 1, 2 .. 10 genes, or using all encountered genes (lightest shade) d. Examples of NOPs for 4 genes.
